# Transcriptome-wide analysis of epitranscriptome and translational efficiency associated with heterosis in maize

**DOI:** 10.1093/jxb/erab074

**Published:** 2021-02-19

**Authors:** Jin-Hong Luo, Min Wang, Gui-Fang Jia, Yan He

**Affiliations:** 1 MOE Key Laboratory of Crop Heterosis and Utilization, National Maize Improvement Center, College of Agronomy and Biotechnology, China Agricultural University, Beijing 100094, China; 2 Synthetic and Functional Biomolecules Center, Beijing National Laboratory for Molecular Sciences, Key Laboratory of Bioorganic Chemistry and Molecular Engineering of Ministry of Education, College of Chemistry and Molecular Engineering, Peking University, Beijing 100871, China; 3 Oklahoma State University, USA

**Keywords:** Heterosis, maize, mRNA, post-transcriptional regulation, RNA m^6^A, translational efficiency

## Abstract

Heterosis has been extensively utilized to increase productivity in crops, yet the underlying molecular mechanisms remain largely elusive. Here, we generated transcriptome-wide profiles of mRNA abundance, m^6^A methylation, and translational efficiency from the maize F_1_ hybrid B73×Mo17 and its two parental lines to ascertain the contribution of each regulatory layer to heterosis at the seedling stage. We documented that although the global abundance and distribution of m^6^A remained unchanged, a greater number of genes had gained an m^6^A modification in the hybrid. Superior variations were observed at the m^6^A modification and translational efficiency levels when compared with mRNA abundance between the hybrid and parents. In the hybrid, the vast majority of genes with m^6^A modification exhibited a non-additive expression pattern, the percentage of which was much higher than that at levels of mRNA abundance and translational efficiency. Non-additive genes involved in different biological processes were hierarchically coordinated by discrete combinations of three regulatory layers. These findings suggest that transcriptional and post-transcriptional regulation of gene expression make distinct contributions to heterosis in hybrid maize. Overall, this integrated multi-omics analysis provides a valuable portfolio for interpreting transcriptional and post-transcriptional regulation of gene expression in hybrid maize, and paves the way for exploring molecular mechanisms underlying hybrid vigor.

## Teaser

A maize F_1_ hybrid and its parental lines showed dissimilarities of regulatory and heterotic patterns of genes undergoing hierarchical regulation at the mRNA abundance, m^6^A modification, and translational efficiency.

## Introduction

Hybrid vigor, or heterosis, refers to the superior performance of F_1_ hybrids over their parents. In plants, heterotic traits are mainly related to growth rate, biomass, stress tolerance, and seed yield. All these traits are crucial for increasing crop yield. The widespread application of heterosis is one of the landmark innovations of modern agriculture, and breeding hybrids has proved to be one of the most efficient ways to increase grain yield of various crops (Hochholdinger and Baldauf, 2018). Although heterosis has been successfully exploited in crop production, the molecular mechanisms underlying it remain largely elusive. Dominance, overdominance, and epistasis have been proposed as classical genetic explanations for heterosis, but these hypotheses have not been connected to molecular principles and do not provide a molecular basis for heterosis (Birchler *et al.*, 2003, 2010).

The putative molecular mechanisms of heterosis are connected with genomic and epigenetic modifications in hybrids. These modifications, in turn, yield advantages in growth, stress resistance, and adaptability of F_1_ hybrids over their parents due to interactions between alleles of the parental genomes that alter regulatory networks of related genes (Alonso-Peral *et al.*, 2017; Shen *et al.*, 2017). Genetic variation is widely studied to understand the molecular basis of heterosis (Huang *et al.*, 2015; Morris *et al.*, 2016; Jiang *et al.*, 2017; Liu *et al.*, 2020). It is assumed that a combination of different genetic principles might run together to explain hybrid vigor (Swanson-Wagner *et al.*, 2006; Lippman and Zamir, 2007). To better decipher the processes underlying the manifestation of heterosis for various phenotypic traits, multifaceted molecular data have been collected at different regulatory levels including the genome (Huang *et al.*, 2015; Lin *et al.*, 2020; Liu *et al.*, 2020), epigenome (Groszmann *et al.*, 2011; Shen *et al.*, 2012; He *et al.*, 2013; Kawanabe *et al.*, 2016; Shen *et al.*, 2017; Zhu *et al.*, 2017; Lauss *et al.*, 2018; Sinha *et al.*, 2020), transcriptome (Paschold *et al.*, 2012; Baldauf *et al.*, 2016; Zhu *et al.*, 2016; Alonso-Peral *et al.*, 2017; Shen *et al.*, 2017; Shao *et al.*, 2019; Sinha *et al.*, 2020), proteome (Hoecker *et al.*, 2008), and metabolome (Romisch-Margl *et al.*, 2010). However, to date we still lack, for any species, fundamental knowledge of how post-transcriptional activities are involved in heterosis.

Modification of the nucleotides of mRNA adds extra information that is not encoded in the mRNA or DNA sequence. The emerging field of epitranscriptomics studies where modified nucleotides are present in mRNA, how they are positioned, read and removed (by ‘writers’, ‘readers’, and ‘erasers’, respectively), and how they may regulate RNA metabolism (Meyer and Jaffrey, 2017; Roignant and Soller, 2017; Roundtree *et al.*, 2017a; Yang *et al.*, 2018; Shen *et al.*, 2019; Yue *et al.*, 2019). *N*^6^-methyladenosine (m^6^A) is the most prevalent covalent modification in mRNA and long non-coding RNA (Dominissini *et al.*, 2012; Meyer *et al.*, 2012). Dynamic m^6^A modification has been implicated in a wide range of RNA metabolic processes, including RNA stability (Wang *et al.*, 2014; Shi *et al.*, 2017; Huang *et al.*, 2018), translation (Meyer *et al.*, 2015; Wang *et al.*, 2015; Li *et al.*, 2017; Shi *et al.*, 2017; Slobodin *et al.*, 2017; Meyer, 2018), alternative splicing (Zhao *et al.*, 2014; Haussmann *et al.*, 2016; Lence *et al.*, 2016; Xiao *et al.*, 2016*a*; Bartosovic *et al.*, 2017; Pendleton *et al.*, 2017), secondary structure (Liu *et al.*, 2015; Liu *et al.*, 2017), and nuclear export (Zheng *et al.*, 2013; Roundtree *et al.*, 2017*b*). In plants, many studies have recently shown that m^6^A modification plays important roles in regulating development (Zhong *et al.*, 2008; Bodi *et al.*, 2012; Shen *et al.*, 2016; Ruzicka *et al.*, 2017; Arribas-Hernandez *et al.*, 2018; Scutenaire *et al.*, 2018; Wei *et al.*, 2018; Zhang *et al.*, 2019; Zhou *et al.*, 2019; Luo *et al.*, 2020; Du *et al.*, 2020) and stress tolerance (Martinez-Perez *et al.*, 2017; Anderson *et al.*, 2018; Li *et al.*, 2018; Miao *et al.*, 2020).

Maize is one of the most important crops worldwide. As a cross-pollinating plant, it displays much stronger heterosis than most other crops. In addition, maize has a remarkable degree of structural intraspecific genomic diversity (Springer *et al.*, 2009). These special characteristics have enabled maize to act as a model organism for studying heterosis over the past few decades. In this study, we integrated and compared the profiles of mRNA abundance, m^6^A methylation, and translational efficiency between the maize F_1_ hybrid B73×Mo17 and its two parental lines to study the association of post-transcriptional regulation of gene expression with heterosis. Our results revealed fairly unique heterotic patterns at different regulatory levels, highlighting that transcriptional and post-transcriptional regulation of gene expression make distinct contributions to heterosis in hybrid maize.

## Materials and methods

### Plant material phenotyping

The maize F_1_ hybrid B73×Mo17 and its parental inbred lines B73 and Mo17 were used in this study. All seeds were sterilized by 70% ethanol and 5% sodium hypochlorite solution and rinsed with sterile water. Then seeds were sown in pots with vermiculite and soil (1:1, v/v) in a growth chamber (16 h of light at 28 °C and 8 h dark at 25 °C). Positioning of the F_1_ hybrid and parental plants was randomized every day. After 14 d, aerial tissues were harvested, immediately frozen in liquid nitrogen and stored at −80 °C for subsequent experiments. The other batch of plants (*n*=15) were used to investigate heterotic traits, including plant height and fresh weight. Statistical significance of differences of heterotic traits was determined using Student’s *t*-test.

### Quantification of m^6^A by LC-MS/MS

Two hundred nanograms of mRNA was digested with 1 U Nuclease P1 (Wako) in buffer containing 10% (v/v) 0.1 M CH_3_COONH_4_ (pH 5.3) at 42 °C for 3 h, followed by the addition of 1 U shrimp alkaline phosphatase (NEB) and 10% (v/v) Cutsmart buffer and incubated at 37 °C for 3 h. Then the sample was diluted to 50 μl and filtered through a 0.22 μm polyvinylidene difluoride filter (Millipore). Finally, 10 μl of the solution was used for LC-MS/MS. Nucleosides were separated using reverse-phase ultra-performance liquid chromatography on a C18 column coupled to online mass spectrometry detection using an Agilent 6410 QQQ triple-quadrupole LC mass spectrometer in positive ion mode. The nucleosides were quantified by comparison with the standard curve obtained from pure nucleoside standards run in the same batch as the samples. The ratio of m^6^A/A was calculated based on the calibration curves.

### m^6^A methylated RNA immunoprecipitation

Total RNA was extracted using TRIzol reagent (Thermo Fisher Scientific) and polyadenylated RNA was subsequently isolated with the GenElute mRNA Miniprep Kit (Sigma-Aldrich) according to the manufacturer’s instructions. m^6^A immunoprecipitation was performed using the Magna methylated RNA immunoprecipitation (MeRIP) m^6^A kit (Millipore) following the manufacturer’s protocol. In brief, 27 μg mRNA was fragmented and ethanol precipitated and 0.5 μg RNA was removed as input control. Meanwhile, 30 μl magnetic A/G beads was incubated with 10 μg anti-m^6^A antibody (MABE1006) in 1× immunoprecipitation (IP) buffer for 30 min at room temperature. Then all remaining fragmented mRNA was incubated with the antibody–beads at 4 °C for 2 h with rotation. After being washed three times with 1× IP buffer, bound RNA was eluted from the beads with 100 μl elution buffer twice and then purified with the RNA Clean & Concentrator Kit (Zymo). Both purified sample and input control were used for library construction.

### Polysome profiling

Polysome profiling was performed as previously described (Zhang *et al.*, 2017). Briefly, 2 g tissue was ground and lysed by incubation for 15 min on ice in 5 ml of polysome extraction buffer (PEB; 200 mM Tris–HCl pH 9.0, 200 mM KCl, 35 mM MgCl_2_, 25 mM EGTA, 1% (v/v) Tween 20, 1% (v/v) Triton X-100, 2% (v/v) polyoxyethylene, 5 mM dithiothreitol, 500 μg ml^−1^ heparin, 100 μg ml^−1^ chloramphenicol, and 25 μg ml^−1^ cycloheximide). After centrifuging at 13 200 *g* for 15 min at 4 °C, the supernatant was loaded on top of a 1.7 M sucrose cushion and centrifuged at 246 078 *g* (SW55Ti rotor in a Beckman L-100XP ultracentrifuge) for 3 h at 4 °C. The pellet was washed with RNase-free water and resuspended with 200 μl resuspension buffer (200 mM Tris–HCl pH 9.0, 200 mM KCl, 35 mM MgCl_2_, 25 mM EGTA, 100 μg ml^−1^ chloramphenicol, and 25 μg ml^−1^ cycloheximide). Then the solution was loaded onto a 20–60% sucrose gradient and centrifuged at 204 275 *g* (SW55Ti rotor) for 2 h at 4 °C. The sucrose gradients were monitored and fractionated with a gradient fractionator (Biocomp, Canada). The polysomal RNA fractions were collected and extracted for library construction.

### Library construction and sequencing

Libraries of RNA-seq, m^6^A-seq, and polysome profiling for the F_1_ hybrid were constructed using the NEBNext Ultra II RNA Library Prep Kit (E7770S, NEB) following the manufacturer’s protocol and sequenced on the Illumina HiSeq X Ten platform using 150 bp paired-end sequencing.

### m^6^A-seq data analysis

Sequencing reads were filtered to remove adapter sequences and low-quality reads using Trimmomatic (v0.35) (Bolger *et al.*, 2014) with parameters ILLUMINACLIP:TruSeq3-PE.fa:2:30:10:1:true LEADING:3 TRAILING:3 SLIDINGWINDOW:4:15 MINLEN:30. To reduce mapping bias and for convenience in comparing the F_1_ hybrid and parents, filtered reads from B73 and F_1_ hybrid B73×Mo17 were mapped to the maize B73 reference genome (AGPv4.38) (Jiao *et al.*, 2017), and filtered reads from Mo17 were mapped to the maize Mo17 pseudogenome constructed by substituting maize B73 reference genome (AGPv4.38) with single nucleotide polymorphisms from Mo17 (CAU-1.0) (Sun *et al.*, 2018) using Hisat2 (v2.1.0) (Kim *et al.*, 2015) with parameters −5 1 −3 1 −−dta. m^6^A peaks were identified by MACS2 peak calling software (v2.1.1) (Zhang *et al.*, 2008) with *q*<0.01 and the overlapped peaks between two biological replicates were designated as high confidence m^6^A peaks and used for subsequent analyses. The m^6^A level was defined as fold change of m^6^A peaks from MACS2 output.

### RNA-seq analysis and translational efficiency calculation

For RNA-seq and polysome profiling, sequencing reads were filtered and mapped as m^6^A-seq. The levels of transcription and translation were estimated by calculating fragments per kilobase of transcript per million fragments (FPKM) by the software StringTie v1.3.3 (Pertea *et al.*, 2015) with default parameters. Only the genes with FPKM ≥1 were considered as expressed genes. The translational efficiency was calculated by ‘FPKM (translational level)/FPKM (transcriptional level)’ as reported previously (Lei *et al.*, 2015). Differentially expressed genes were identified by the DESeq2 package (Love *et al.*, 2014) with fold-change ≥1.5, *P*<0.01 between parents and between the hybrid and parents, and non-additive genes in the hybrid were defined with significantly differential expression levels against mid-parent value (MPV) at fold-change ≥1.5 and *P*<0.01. Similarly, differentially translated genes were identified by the Xtail package (v1.1.5) (Xiao *et al.*, 2016*b*) with fold-change ≥1.5, *P*<0.01 between parents and between hybrid and parents, and non-additively translated genes in the hybrid were defined with significantly different translational efficiency against MPV at fold-change ≥1.5 and *P*<0.01.

### Gene ontology analysis

GO analysis were performed using FuncAssociate 3.0 (http://llama.mshri.on.ca/funcassociate/) (Berriz *et al.*, 2009) and GO terms with adjusted *P*<0.001 were defined as significant.

### Definition of *cis* and *trans* regulatory divergence

Unique reads of the F_1_ hybrid were obtained by selecting the alignment records with the ‘NH:i:1’ tag. Single nucleotide polymorphisms between the B73 and Mo17 genomes were identified with Mummer v3.0 (Kurtz *et al.*, 2004) as described previously (Sun *et al.*, 2018). SNPsplit v0.3.4 (Krueger and Andrews, 2016) was used with default parameters to determine the parental origins, and all SNP-containing reads were used for allele-specific expression analyses. *Cis* and *trans* effects were explored as previously described (Bao *et al.*, 2019). Parental gene expression and F_1_ allelic expression were combined to characterize *cis* and *trans* effects. Parental gene expression divergence was defined as *A*, and F_1_ allelic expression divergence as *B*. The genes exhibited F_1_ allelic divergence equivalent to parental gene expression divergence were considered to be caused by only *cis* effects ((i) *A*≠0, *B*≠0, *A*=*B*). If parents showed significant divergence but not F_1_ allelic expression, genes were considered to be caused by only *trans* effects ((ii) *A*≠0, *B*=0, *A*≠*B*). Genes exhibiting F_1_ allelic divergence that significantly diverged from parental gene expression divergence ((iii) *A*≠0, *B*≠0, *A*≠*B*) were considered to be enhancing or compensating, which was dependent on whether the *cis* and *trans* effects were in the same or opposite directions, respectively. When F_1_ allelic expression showed significant divergence but not between parents, genes were considered as fully compensatory ((iv) *A*=0, *B*≠0, *A*≠*B*). Genes belonging to category (iii) and (iv) were combined and defined as both *cis* and *trans* effect. Neither parental gene expression divergence nor F_1_ allelic expression divergence was detected, which was defined as conserved genes ((v) *A*=0, *B*=0, *A*=*B*).

### RT-qPCR

RT-qPCR was performed as described in Duan *et al.* (2017) and Zhang *et al.* (2017). Briefly, RNA from m^6^A-IP, input, and polysome profiling was used for reverse transcription with the PrimeScript^TM^ RT reagent Kit with gDNA Eraser (TaKaRa). RT-qPCR was performed with TB Green Premix Ex Taq (TaKaRa) using a Bio-Rad CFX96 real-time PCR detection system. *Zm00001d034600* and *Zm00001d042939* were used as internal control genes due to their invariant expression among hybrid and two parental lines B73 and Mo17 for all the three levels of mRNA abundance, m^6^A methylation, and translational efficiency according to the sequencing data. All primers used in this study are listed in Supplementary Table S1.

## Results

### Remarkable redistribution of m^6^A epitranscriptome in maize hybrid

**Fig. 1.**
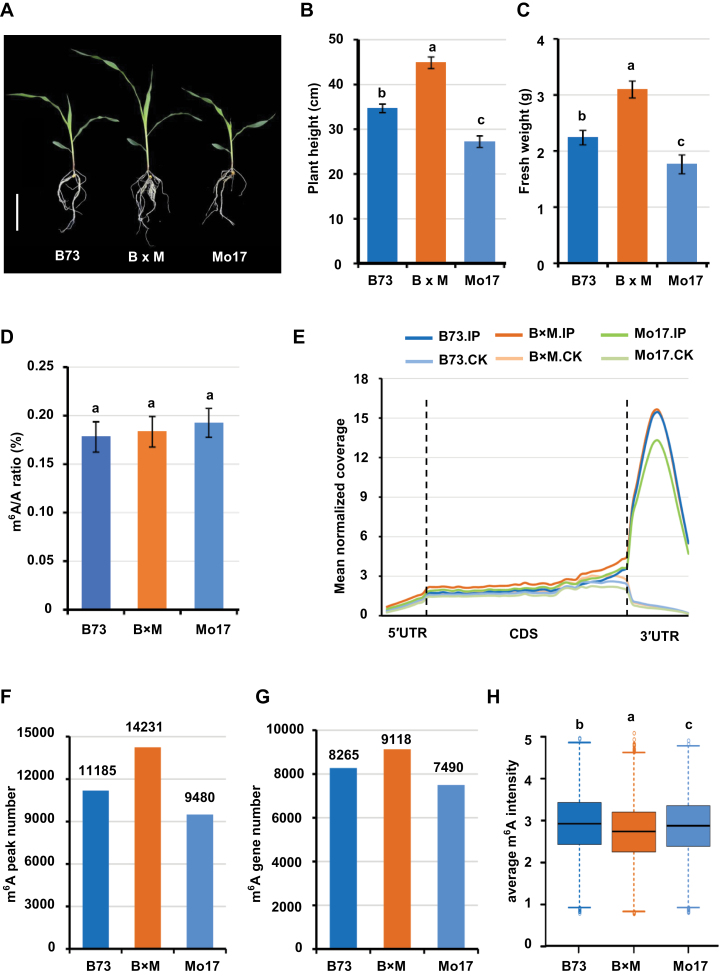
Heterosis of vegetative growth and m^6^A modification in maize F_1_ hybrid seedlings at 14 DAS. (A) Heterotic phenotype of the maize hybrid B73×Mo17 relative to the parental lines B73 and Mo17. Bar: 20 cm. (B–H) Comparison between hybrid and parental lines of plant height (B), fresh weight (C), total m^6^A abundance (D), m^6^A peak configuration (E), total number of m^6^A peaks (F), total number of m^6^A-modified genes (G), and average m^6^A intensity (H). IP, m^6^A peaks; CK, negative peaks. Duncan’s analysis was employed to test statistical significance between hybrid and parental lines. Different letters on the graphs indicate significant differences at *P*<0.05. Error bars indicate the standard deviation with 15 biological replicates in (B, C) and three biological replicates in (D). B×M, the hybrid B73×Mo17; CDS, coding sequence.

To better understand the molecular mechanisms underlying heterosis in maize, we used the maize inbred lines B73 and Mo17, and their F_1_ hybrid B73×Mo17 as research targets. Significant growth vigor in the F_1_ hybrid was observed at the early seedling stage 14 d after sowing (DAS) (Fig. 1A). We compared heterotic phenotypes for biomass, plant height, and fresh weight. The plant height of the F_1_ hybrid was 45.0% and 29.4% greater than the mid-parent value (MPV) and better parent value (BPV), respectively (Fig. 1B). The fresh weight of the F_1_ hybrid was 54.8% and 38.3% larger than the MPV and BPV, respectively (Fig. 1C). These results clearly indicate that the maize F_1_ hybrid B73×Mo17 plants at 14 DAS displayed vigorous heterosis, and therefore aerial tissues at 14 DAS were collected as research material for the subsequent analyses.

To explore whether epitranscriptomic regulation of gene expression is associated with heterosis, we firstly measured the m^6^A/A ratio of purified mRNA by using LC-MS/MS to show the global abundance of m^6^A modification *in planta*. As shown in Fig. 1D, no significant difference was observed for the m^6^A/A ratio between the F_1_ hybrid and the two parental lines, suggesting that the global m^6^A methylation abundance remains relatively stable in the hybrid.

To gain more insight into the regulation of m^6^A methylation in gene expression in the hybrid, we generated transcriptome-wide integrated maps of mRNA abundance, m^6^A methylation, and translational efficiency by conducting input RNA sequencing (RNA-seq), m^6^A RNA immunoprecipitation sequencing (m^6^A-seq) (Dominissini *et al.*, 2012; Meyer *et al.*, 2012), and polysome profiling (Juntawong *et al.*, 2014; Zhang *et al.*, 2017) in the F_1_ hybrid for two independent biological replicates. Critically, all plant material used to produce these datasets from hybrid and parental lines was grown at the exact same time and under the same conditions. It should be noted that the same datasets including RNA-seq, m^6^A-seq, and polysome profiling from the two parental lines, B73 and Mo17, have been published in our recent study to interpret natural variation in m^6^A modification (Luo *et al.*, 2020). Two biological replicates showed a high degree of correlation for RNA-seq, m^6^A-seq, and polysome profiling data in the hybrid (see Supplementary Fig. S1) and in the parental lines (Luo *et al.*, 2020). Moreover, for another two independent biological replicates the levels of mRNA abundance, m^6^A methylation, and translational efficiency for eight randomly selected genes were examined by RT-qPCR analysis, and were largely consistent with the sequencing data (Supplementary Figs S2–S4). These results corroborated the reliability of our data and allowed us to conduct further statistical analyses.

Similar to B73 and Mo17, m^6^A peaks in the hybrid were primarily enriched in the 3′-untranslated region (UTR; ~69.9%) and in the vicinity of the stop codon (~21.1%; defined as a 200-nt window centered on the stop codon), but were less present in coding sequences (CDS; ~3.2%), near start codons (~0.2%; defined as a 200-nt window centered on the start codon), in the 5′UTR (~0.6%), and in the spliced intronic regions (~5.1%; Fig. 1E; Supplementary Fig. S5), indicating that the overall configuration of m^6^A is unchanged in the hybrid. Interestingly, a much greater number of m^6^A peaks (*n*=14 231) were identified in the hybrid in comparison with the parental line B73 (*n*=11 185) and Mo17 (*n*=9480) (Fig. 1F), although the number of genes containing multiple m^6^A peaks was comparable between the hybrid and parental lines (Supplementary Fig. S6). Accordingly, the number of genes containing m^6^A peaks (*n*=9118; Supplementary Table S2) was greater in the hybrid than in the parental B73 (*n*=8265) and Mo17 (*n*=7490) lines (Fig. 1G). Interestingly, the average intensity of m^6^A peaks was less in the hybrid (Fig. 1H). These results suggest that m^6^A modification exhibits both common and unique features in the F_1_ hybrid in comparison with its parents.

### Distinct regulatory patterns at the levels of mRNA abundance, m^6^A modification, and translational efficiency between hybrid and parents

**Fig. 2.**
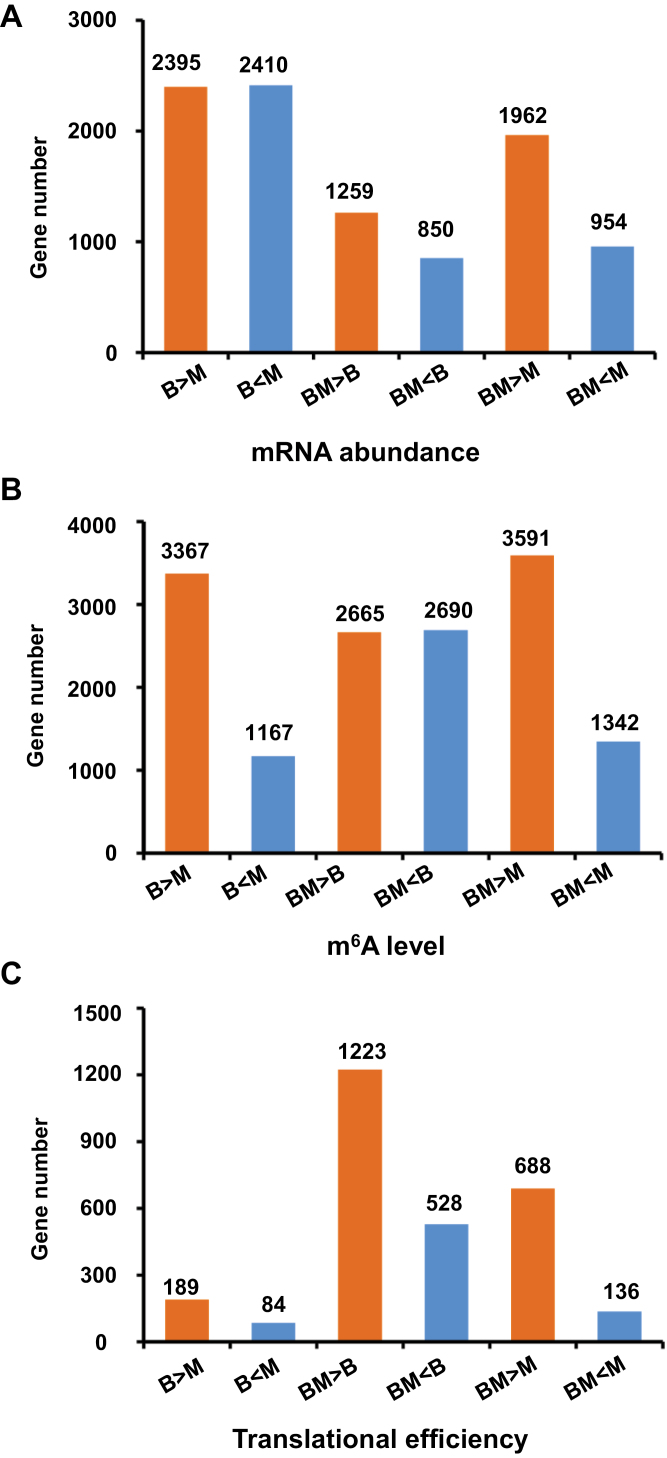
Differential genes among hybrid and parents at the levels of mRNA abundance, m^6^A modification and translational efficiency. (A–C) Differential genes at the mRNA abundance level (A), m^6^A modification (B), and translational efficiency (C) were identified on the basis of six pairwise comparisons among the hybrid and parents. The number of differential genes, which were designated with false discovery rate (FDR) <0.01 and fold change ≥1.5, are shown above each bar. B, B73; M, Mo17; BM, B73×Mo17.

To ascertain conservation and divergence of mRNA abundance, m^6^A modification, and translational efficiency in the hybrid relative to the parental lines, six pairwise comparisons were performed per regulatory layer. Intriguingly, fairly distinct patterns in the pairwise comparisons between hybrid and parents and between parents were observed among the three regulatory layers. At the mRNA abundance level, the number of differentially expressed genes in parent–hybrid comparisons was substantially less than in parent–parent comparisons, with 4805 genes between parents in comparison with 2109 and 2916 genes in the hybrid relative to B73 and Mo17, respectively (Fig. 2A). At the m^6^A level, the number of genes with differential degrees of modification in parent–hybrid comparisons was approximately equal to that in parent–parent comparisons, with 4534 genes between parents in comparison with 5355 and 4933 genes in the hybrid relative to B73 and Mo17, respectively (Fig. 2B). At the translational efficiency level, the number of genes with differential translational efficiency in parent–hybrid comparisons was much greater than in parent–parent comparisons, where there were only 273 genes differing between parents in comparison with 1751 and 824 genes in the hybrid relative to B73 and Mo17, respectively (Fig. 2C). Together, these results indicate that transcriptional and post-transcriptional regulation of gene expression show distinct modes between the parents and hybrid.

### Distinct heterotic patterns at the levels of mRNA abundance, m^6^A modification, and translational efficiency in hybrid

**Fig. 3.**
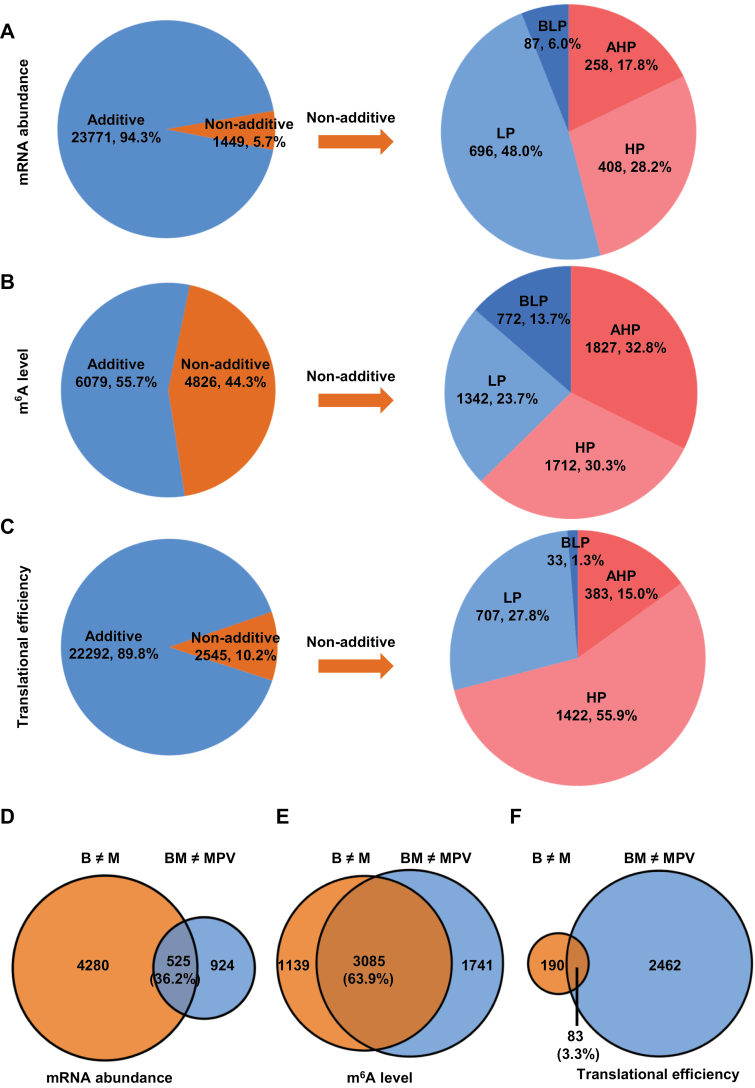
Heterotic patterns at the levels of mRNA abundance, m^6^A modification, and translational efficiency in the hybrid. (A–C) Additive and non-additive variation and subdivided patterns of non-additive variation (AHP, above higher parent; HP, high parent; LP, low parent; BLP, below lower parent) at the levels of mRNA abundance (A), m^6^A modification (B), and translational efficiency (C) in the hybrid. The numbers and percentages of genes exhibiting additive and non-additive variation as well as subdivided patterns of non-additive variation are displayed. (D–F) Contribution to non-additive variation in the hybrid by the divergence of parents at the levels of mRNA abundance (D), m^6^A modification (E), and translational efficiency (F). B≠M, significant difference between parents; BM≠MPV, significant difference between hybrid and MPV. B, B73; BM, B73×Mo17; M, Mo17.

Non-additive gene action has been regarded as a specific expression pattern in hybrids and could potentially be responsible for generating heterotic phenotypes (Li *et al.*, 2015; Zhao *et al.*, 2019). We designated genes in the F_1_ hybrid with a significant difference from MPV (*P*<0.01; false discovery rate (FDR)<0.01) as non-additive genes at each of the mRNA abundance, m^6^A modification, and translational efficiency levels. Strikingly, we observed that the percentage and number of non-additive genes were extraordinarily different at each of the three regulatory layers in the hybrid. In particular, 44.3% of m^6^A-modified genes (*n*=4826) were non-additive (Fig. 3B; Supplementary Table S3), and this percentage was far more than for non-additive genes at the mRNA abundance (5.7%, *n*=1449; Fig. 3A; Supplementary Table S4) and translational efficiency level (10.2%, *n*=2545; Fig. 3C; Supplementary Table S5). The large percentage of non-additive m^6^A modification implies its likely active involvement in heterosis.

To better visualize non-additive genes in the hybrid, we divided the non-additive genes into four categories, including above higher parent (AHP; the value in the hybrid is above the higher parent), high parent (HP; the value in the hybrid is similar to the higher parent), low parent (LP; the value in the hybrid is similar to the lower parent), and below lower parent (BLP; the value in the hybrid is below the lower parent) (Birchler *et al.*, 2003; Springer and Stupar, 2007). Again, we observed fairly distinct patterns of non-additive genes at each of the three regulatory layers. Different from the mRNA abundance level, at which the number and proportion of up-regulated genes (*n*=666, 46.0%) were moderately lower than those of down-regulated genes (*n*=783, 54.0%), the numbers and proportions of up-regulated genes at both the m^6^A modification (*n*=3539, 63.1%) and translational efficiency level (*n*=1805, 70.9%) were much greater than those of down-regulated genes (*n*=2114, 36.9%, and *n*=740, 29.1% for m^6^A modification and translational efficiency, respectively) (Fig. 3A–C), suggesting that increased m^6^A modification and translational efficiency may be critically involved in heterosis.

To characterize parent-of-origin effects on gene activity, we compared parental and heterotic variances at the levels of mRNA abundance, m^6^A methylation, and translational efficiency. Interestingly, we found that parental variances in m^6^A methylation contributed more to heterotic variances relative to mRNA abundance and translational efficiency. In detail, 63.9% of non-additive m^6^A-modified genes (Fig. 3E, *n*=3085) could be explained from parental variances, whereas only 36.2% (Fig. 3D, *n*=525) and 3.3% (Fig. 3F, *n*=83) could be explained at the mRNA and translational efficiency levels, respectively. Together, these results clearly indicate that transcriptional and post-transcriptional regulation of gene expression participate differently in the formation of heterosis in the maize hybrid.

### Cooperative regulation of mRNA abundance, m^6^A modification, and translational efficiency in hybrid

**Fig. 4.**
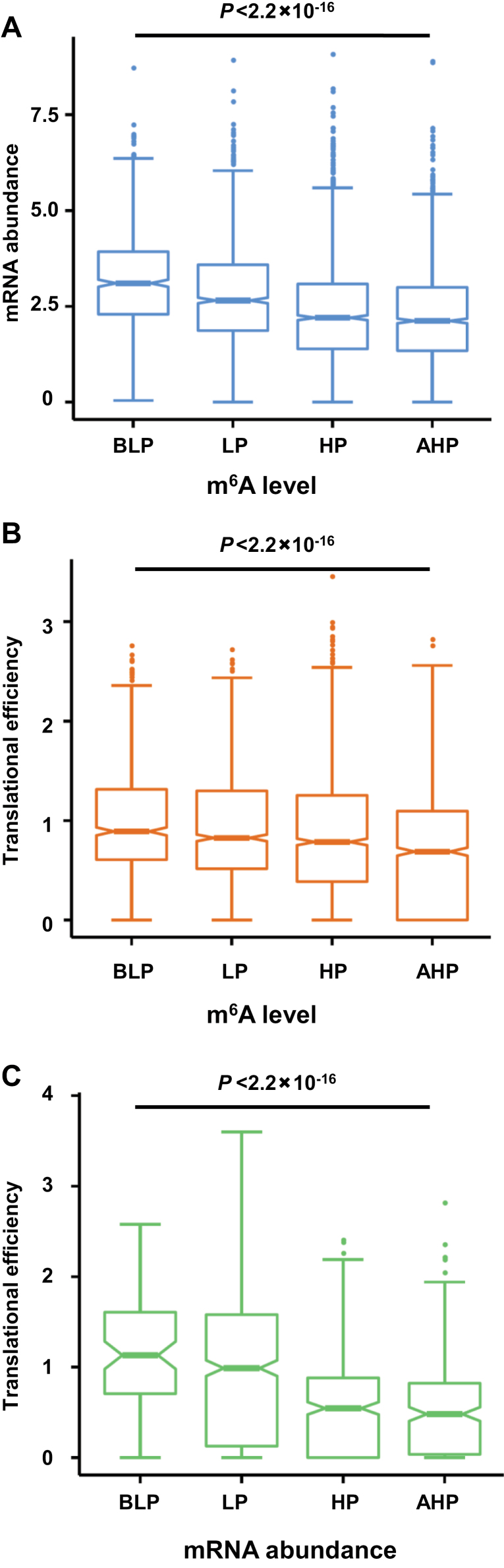
Relationship of gene expression at the levels of mRNA abundance, m^6^A modification, and translational efficiency in the hybrid. (A) Correlation between mRNA abundance and different patterns of non-additively m^6^A-modified genes. (B) Correlation between translational efficiency and different patterns of non-additively m^6^A-modified genes. (C) Correlation between translational efficiency and different patterns of non-additively transcribed genes. The *P*-value was calculated using the Kruskal–Wallis test.

The key roles of m^6^A in epitranscriptomic regulation of gene expression prompted us to investigate its effects on mRNA abundance and translational efficiency. As shown in Fig. 4A, genes in the groups HP and AHP categorized by m^6^A level showed a decreased level of mRNA abundance compared with genes in the LP and BLP groups, suggesting that m^6^A modification may be actively involved in mRNA decay in the hybrid. Likewise, genes in the HP and AHP groups categorized by m^6^A level exhibited a tendency for decreased level of translational efficiency compared with genes in the LP and BLP groups (Fig. 4B), suggesting that the high degree of m^6^A modification may also attenuate translational efficiency in the hybrid. Moreover, genes in the HP and AHP groups categorized by mRNA abundance displayed a much lower level of translational efficiency than gene in the LP and BLP groups (Fig. 4C), suggesting that gene transcription and translation activity are negatively correlated in the hybrid. Together, these results suggest that mRNA abundance, m^6^A modification, and translational efficiency may cooperatively maintain the homeostasis status of non-additive gene expression in the hybrid.

### Distinct enrichment of biological pathways coordinated at the levels of mRNA abundance, m^6^A modification, and translational efficiency in hybrid

**Fig. 5.**
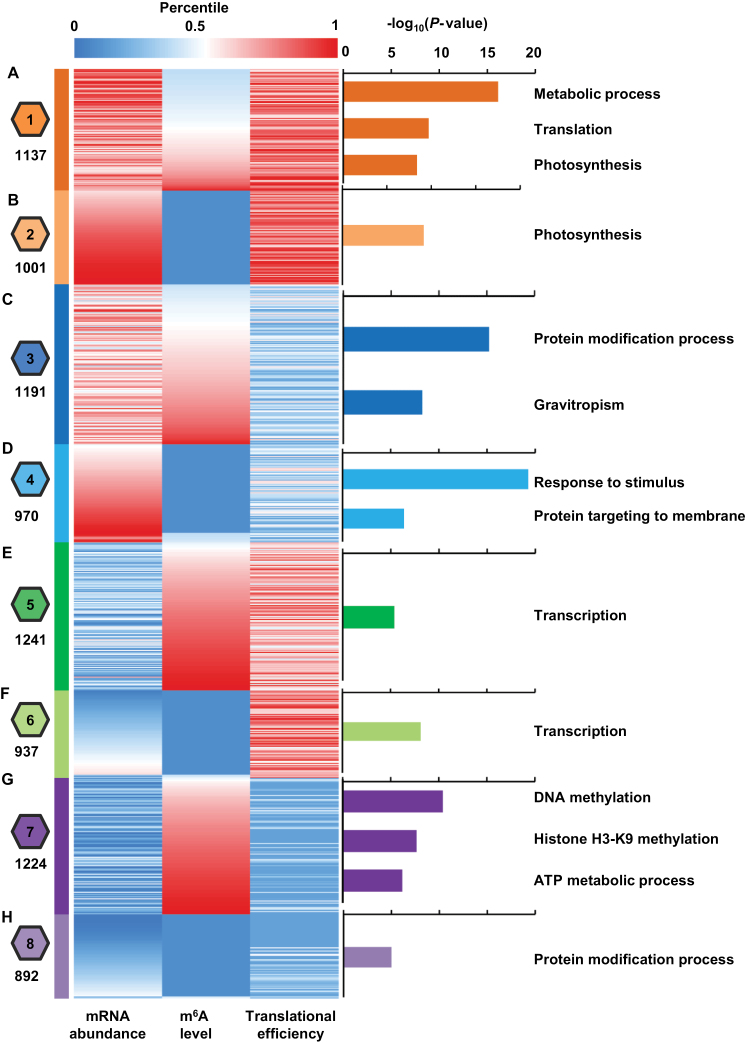
Distinct enrichment of biological pathways coordinated at the levels of mRNA abundance, m^6^A modification, and translational efficiency in the hybrid. (A–H) Non-additive genes characterized by mRNA abundance, m^6^A modification, and translational efficiency were compiled together and subject to *k*-means clustering analysis. Each level of mRNA abundance, m^6^A intensity, and translational efficiency was converted to percentiles using the empirical cumulative distribution function. The color indicates the relative level of mRNA abundance, m^6^A intensity, and translational efficiency. A total of eight clusters were identified and the number of genes in each cluster is shown at the left. Significantly enriched gene ontology (GO) terms were identified by FuncAssociate 3.0 (permutation-based corrected *P*<0.001) and shown at the right. The details of all significantly enriched GO terms are listed in Supplementary Table S6.

To investigate enrichment of biological pathways coordinated by the three different regulatory layers, we performed a *k*-means clustering analysis to group all the non-additive genes defined from all three regulatory layers into eight classes based on levels of mRNA abundance, m^6^A modification, and translational efficiency (Fig. 5A–H; see ‘Materials and methods’). We then conducted a Gene Ontology (GO) term enrichment analysis across all the different clusters (see Supplementary Table S6). Interestingly, we observed some common but mostly unique biological pathways enriched in each individual cluster (Fig. 5A–H).

In cluster 1, which was signified by a high level of mRNA abundance, median to high level of m^6^A modification, and high level of translational efficiency, the three most significantly enriched groups were metabolic process, translation, and photosynthesis (Fig. 5A). Similar with a high level of mRNA abundance and translational efficiency, but differing by a low level of m^6^A modification, cluster 2 only contained one group, photosynthesis (Fig. 5B). The shared group of photosynthesis between cluster 1 and cluster 2 suggests that high activity of transcription and translation for genes involved in the photosynthesis pathway is not affected by m^6^A modification.

In cluster 3, which was signified by a high level of mRNA abundance and m^6^A modification, but low level of translational efficiency, the two most significantly enriched groups were protein modification process and gravitropism (Fig. 5C). The opposite patterns of transcription and translation suggests that the high transcriptional activity of genes involved in these biological processes may be attenuated by decreased translational activity via a high degree of m^6^A modification. Cluster 4 was signified by a high level of mRNA abundance, but low levels of m^6^A modification and translational efficiency (Fig. 5D). The enriched groups included response to stimulus and protein targeting to membrane (Fig. 5D). Interestingly, the response to stimulus pathway represented the most significant group (*P*<5.4×10^–20^) and contained the maximum number of genes (*n*=278) identified in all the clusters. The opposite patterns of transcription and translation indicates that a high level of mRNA of genes involved in response to stimulus pathway may be substantially attenuated by decreased translational activity, and this attenuation is likely not dependent on m^6^A modification.

Cluster 5 and cluster 6 were signified by a low level of mRNA abundance and high level of translational efficiency, but differed in the level of m^6^A modification (Fig. 5E, F). The same but only pathway enriched in these two clusters was transcription (Fig. 5E, F), suggesting that the reduced transcription of genes involved in the transcription pathway may be compensated by increased translational activity in the hybrid, whereas this increase is not likely dependent on m^6^A modification. Cluster 7 was signified by a high level of m^6^A modification, and contained three groups, DNA methylation, histone H3–K9 methylation, and ATP metabolic pathways (Fig. 5G). Cluster 8 exhibited low levels at all three regulatory layers (Fig. 5H). Together, the specific enrichments identified in all eight clusters suggest that genes involved in various biological pathways may be subject to hierarchical coordination in terms of three regulatory layers.

### Distinct *cis* and *trans* regulatory patterns at the levels of mRNA abundance, m^6^A modification, and translational efficiency in hybrid

Previous studies have reported that parental alleles show biased expression in maize hybrids (Stupar and Springer, 2006; Guo *et al.*, 2008). To understand how parental alleles contribute to differential gene expression in three different regulatory layers, we performed allelic bias analysis in the hybrid using single nucleotide polymorphisms (SNPs) between parental lines B73 and Mo17. Allele-specific sequencing reads discriminated by SNPs were utilized to evaluate allelic bias in the hybrid. To ensure accuracy and reliability, only SNPs identified with a significant allele-specific bias at a *P*-value cutoff below 0.01 in the hybrid were used in further analyses. Using this criterion, 973, 41, and 30 genes were identified with allelic bias for mRNA abundance, m^6^A modification, and translational efficiency, respectively (Table 1). Discrimination of the differential allelic effects based on the direction of allelic bias in the hybrid exhibited no obvious bias toward either B73 or Mo17 (Table 1), indicating that two parental genomes may contribute equally to the mRNA abundance, m^6^A modification, and translational efficiency in the maize hybrid.

**Table 1.** Genes with allelic bias at the levels of mRNA abundance, m^6^A modification, and translational efficiency in the hybrid

	Total	Total B^a^:M^a^>1	B^a^:M^a^<1
mRNA abundance	973	462	511
m^6^A modification	41	19	22
Translational efficiency	30	16	14

Only genes identified with a significant allelic bias at a *P*-value cutoff of 0.01 were included. B^a^, B73 allele; M^a^, Mo17 allele.

Gene expression is regulated through the interactions of *cis* and *trans* regulatory elements. *Cis* regulatory elements are short DNA sequences containing specific binding sites for *trans* factors to control expression of their associated genes (Bao *et al.*, 2019). Based on the statistical tests of parental and F_1_ alleles, genes were assigned to one of four regulatory categories, namely *cis* only, *trans* only, *cis* and *trans*, and conserved genes (Table 2). Although the category of conserved genes represented the majority in all three regulatory layers, the percentage of genes in the other three categories displayed substantial differences (Table 2). In particular, a large number of *trans*-only genes (*n*=988, 25.7%) were observed at the level of m^6^A modification (Table 2), suggesting that the *trans* effect may play a greater role than *cis* or *cis* and *trans* effects in defining differentially m^6^A-modified genes in the F_1_ hybrid.

**Table 2.** Number and percentage of genes with a *cis*- or *trans*-effects only, with both *cis*- and *trans*-effects, or conserved genes at the levels of mRNA abundance, m^6^A modification, and translational efficiency

	mRNA abundance (*n* (%))	m^6^A modification (*n* (%))	Translational efficiency (*n* (%))
*Cis* only	487 (4.1)	16 (0.4)	1 (0.0)
*Trans* only	446 (3.7)	988 (25.7)	153 (1.1)
*Cis* and *trans*	377 (3.2)	13 (0.3)	29 (0.2)
Conserved	10 654 (89.1)	2826 (73.5)	13 382 (98.7)

## Discussion

Many previous studies in maize have provided interesting insights into heterotic patterns at epigenomic fields, including DNA methylation (Shen *et al.*, 2012; Kawanabe *et al.*, 2016; Lauss *et al.*, 2018; Sinha *et al.*, 2020), histone modification (He *et al.*, 2013; Zhu *et al.*, 2017), and sRNA abundance (Groszmann *et al.*, 2011; Greaves *et al.*, 2016; Crisp *et al.*, 2020). However, the recognized regulation by the epigenome of gene expression primarily occurs at the level of transcription. Therefore, we basically know nothing about whether post-transcriptional regulation of gene expression contributes to heterosis. If it does, what is the regulatory manner and how is it different from transcription? Meanwhile, it is well known that gene transcription cannot entirely determine protein abundance due to several post-transcriptional events such as alternative splicing, mRNA modification, translational efficiency, proper protein folding, and post-translational modification (de Sousa Abreu *et al.*, 2009; Vogel and Marcotte, 2012; Wang *et al.*, 2015; Vitrinel *et al.*, 2019). In the present work, we conducted the integrated measurement of mRNA abundance, m^6^A modification, and translational efficiency in a maize F_1_ hybrid and its parental lines, and aimed to reveal the first genome-wide pattern of post-transcriptional regulation of gene expression underlying heterosis. Our results revealed remarkable dissimilarities of regulatory and heterotic patterns among mRNA abundance, m^6^A modification, and translational efficiency. Moreover, we discovered that genes participating in different biological pathways may undergo hierarchical regulation, which was coordinated by discrete combinations of three regulatory layers.

Serving as an epitranscriptomic layer of gene regulation, dynamic m^6^A modification has been demonstrated to play vital roles in a wide range of RNA metabolic processes (Roignant and Soller, 2017; Yang *et al.*, 2018; Shen *et al.*, 2019; Huang *et al.*, 2020). We found that although the global abundance and configuration of m^6^A were comparable between hybrid and parents, the number of genes harboring m^6^A sites was increased in the hybrid (Fig. 1). However, an equivalent global abundance but increased number of m^6^A-modified genes seems controversial. This concern is well reconciled by the fact that the average intensity of m^6^A peaks was reduced in the F_1_ hybrid compared with the two parental lines, suggesting that m^6^A modification may post-transcriptionally fine-tune expression of a greater number of genes in the hybrid (Fig. 1). This provides the first hint of the prospective importance of m^6^A modification in the formation of heterosis. Secondly, the percentage of non-additive m^6^A-modified genes is extraordinarily higher than that of mRNA abundance and translational efficiency (Fig. 3). It has been recognized that non-additive gene activity can be the major force driving the formation of heterosis (Li *et al.*, 2015; Zhao *et al.*, 2019). Therefore, although the exact biological effect of m^6^A sites on each individual gene must vary gene-by-gene, the active involvement of m^6^A modification in heterosis is hypothetically conceivable.

Numerous previous studies have shown that the transcription of a series of stimulus-responsive genes was up-regulated in hybrids (Groszmann *et al.*, 2015; Yang *et al.*, 2015). Consistently, we found that the pathway of response to stimulus was strikingly enriched in the group exhibiting an increased level of mRNA. However, surprisingly, this group of genes also displayed reduced translational efficiency, indicating that although up-regulated for mRNA abundance, the cellular activity of these stress-responsive genes might be substantially attenuated at the translation level (Fig. 5). This raises two intriguing questions of how this antagonistic pattern of up-regulated transcription but down-regulated translation is fulfilled and to what extent it contributes to heterosis. Our previous study has indicated that the excessive extent of m^6^A modification may inhibit the translational status in maize (Luo *et al.*, 2020), and therefore we originally speculated that m^6^A modification may play a role in this process. However, this assumption was principally ruled out because the level of m^6^A modification in this group was fairly low, meaning that the other alternative post-transcriptional process must operate specifically to reduce translational efficiency of these stress-responsive genes. In addition, many previous studies have suggested that the increased transcription of stress-responsive genes may be attributed to enhanced stress tolerant in the hybrid (Groszmann *et al.*, 2015; Yang *et al.*, 2015). If this is true, why does it exhibit the suppression of translational efficiency? We hypothesize one likelihood is that decreased translational efficiency may constrain the production of proteins encoded by these stress-responsive genes, consequently maintaining the homeostasis of gene activity to fulfil the biological balance between plant growth and stress tolerance. This trade-off phenomenon has been well documented in many important early works (Chapin, 1991; Skirycz *et al.*, 2010; Skirycz and Inze, 2010). In this scenario, the increased transcription of stress-responsive genes may be beneficial in the resilience of plants to environmental stress. However, the attenuated translation would likely optimize fitness costs associated with defense to promote plant growth.

Unlike the stress-responsive pathway, genes linked with photosynthesis, metabolic, translation, and nucleosome assembly pathways showed constitutively high levels of mRNA abundance and translational efficiency (Fig. 5). Apparently, these pathways have housekeeping functions, and the superior activity is critically needed for the rapid growth and development of the hybrid plant. In contrast, genes involved in the transcription pathway showed contrasted patterns with a low level of mRNA abundance but a high level of translational efficiency (Fig. 5). Interestingly, genes related to the establishment and maintenance of the epigenome, i.e. DNA methylation and histone modification, displayed a high level of m^6^A modification, but low levels of both mRNA abundance and translational efficiency, implying that there may exist some types of crosstalk between the epigenome and the epitranscriptome, which has been recently suggested in human cells (Huang *et al.*, 2019) and Arabidopsis (Shim *et al.*, 2020) (Fig. 5). Therefore, if and how this crosstalk contributes to the formation of heterosis deserves further investigation.

In sum, we describe the first parallel analysis of mRNA abundance, m^6^A modification, and translational efficiency profiles in a hybrid and its parental lines. We found many unique features of m^6^A modification and translational efficiency in the hybrid when compared with mRNA abundance, and demonstrated that post-transcriptional controls on gene expression may actively contribute to heterosis in maize. We further identified that gene expression of different biological pathways was under hierarchical control, which was coordinated by three regulatory layers, highlighting that transcriptional and post-transcriptional controls on gene action run together to establish the molecular basis of heterosis. Therefore, our study adds a new dimension to the exploration of core mechanisms underlying heterosis.

## Supplementary data

The following supplementary data are available at *JXB* online.

Fig. S1. The repeatability between two biological replicates for RNA-seq data, m^6^A-seq data, and polysome profiling data in the hybrid.

Fig. S2. RT-qPCR validation of eight genes at the mRNA level in F_1_ hybrid B73×Mo17 and its two parental lines, B73 and Mo17.

Fig. S3. RT-qPCR validation of eight genes at the m^6^A level in F_1_ hybrid B73×Mo17 and its two parental lines, B73 and Mo17.

Fig. S4. RT-qPCR validation of selected eight genes at the level of translational efficiency in F_1_ hybrid B73×Mo17 and its two parental lines, B73 and Mo17.

Fig. S5. Pie-chart depicting the percentage of m^6^A peaks within six transcript segments in the hybrid.

Fig. S6. Comparison of gene numbers containing multiple m^6^A peaks between hybrid and parents.

Table S1. The list of primers used in the study.

Table S2. The list of m^6^A-modified genes showing peak summit locations, mRNA abundance, m^6^A level, and translational efficiency in the maize F_1_ hybrid B73×Mo17.

Table S3. The heterotic types of non-additive genes at the level of m^6^A modification in the maize F_1_ hybrid B73×Mo17.

Table S4. The heterotic types of non-additive genes at the level of mRNA abundance in the maize F_1_ hybrid B73×Mo17.

Table S5. The heterotic types of non-additive genes at the level of translational efficiency in the maize F_1_ hybrid B73×Mo17.

Table S6. Significantly enriched Gene Ontology (GO) terms for all eight clusters identified in Fig. 5.

erab074_suppl_Supplementary-Figures-S1-S6

erab074_suppl_Supplementary-Tables-S1-S6

## Data Availability

All the raw data for F_1_ hybrid B73×Mo17 have been deposited in the Gene Expression Omnibus (GEO; https://www.ncbi.nlm.nih.gov/geo) under accession number GSE155947. The raw data for parental lines B73 and Mo17 have been published and under accession number GSE124543.
