# Supplementary material for: Transcriptome-wide analysis of epitranscriptome and translational efficiency associated with heterosis in maize
Source: J Exp Bot. 2021 Feb 19;72(8):2933–46. doi: 10.1093/jxb/erab074 (PMC8023220; doi:10.1093/jxb/erab074)
Supplement: erab074_suppl_Supplementary-Figures-S1-S6 [file erab074_suppl_supplementary-figures-s1-s6.pdf]

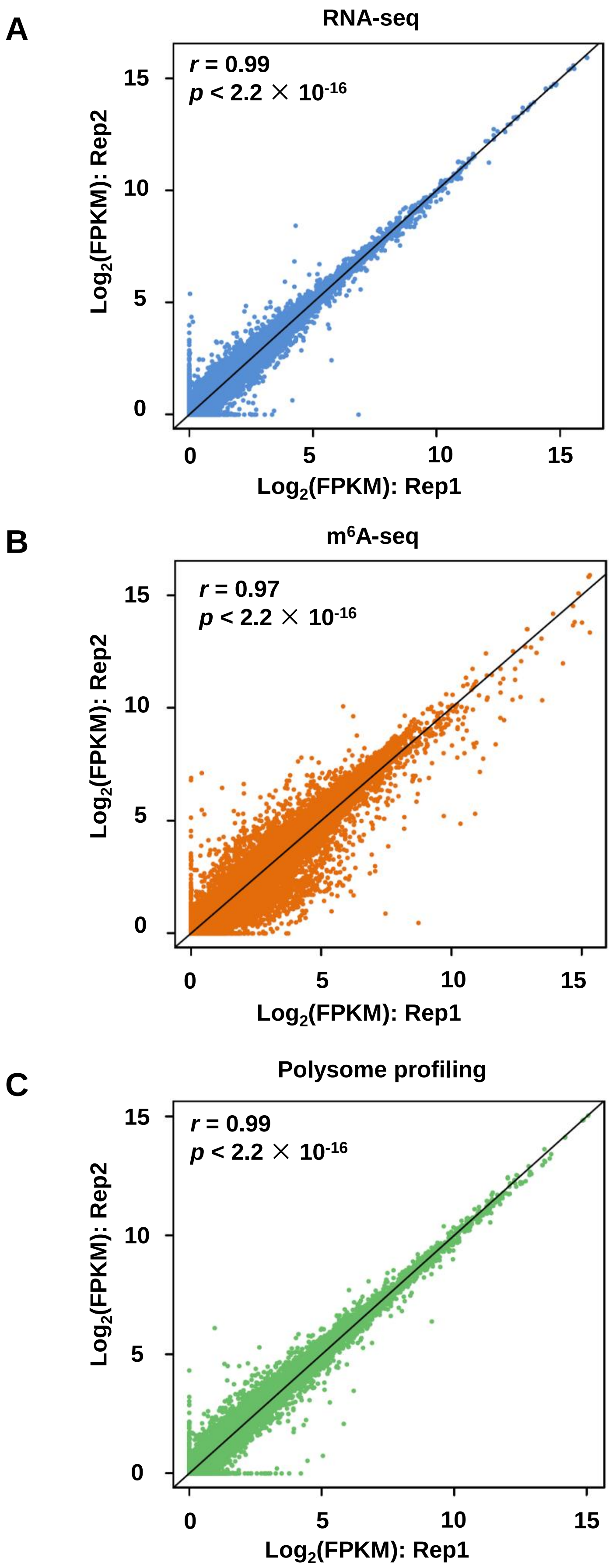

Figure S1. The repeatability between two biological replicates for RNA-seq data (A), m^6A-seq data (B) and polysome profiling data (C) in hybrid. The Pearson correlation coefficient (*r*) and *p*-values are shown in the top left corner.

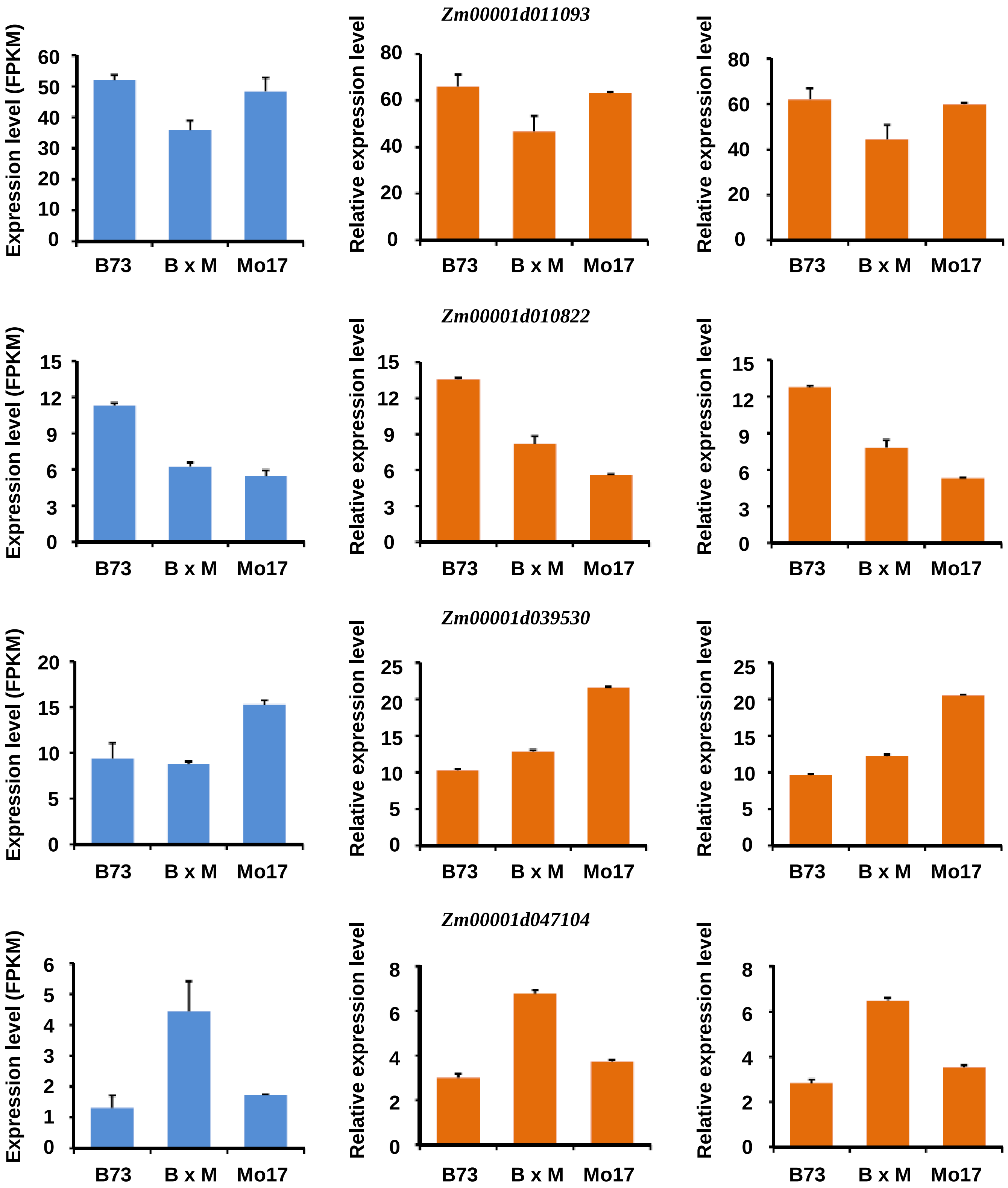

Zm00001d011093
Zm00001d010822
Zm00001d039530
Zm00001d047104
Expression level (FPKM)
Relative expression level
B73
B x M
Mo17

Continued

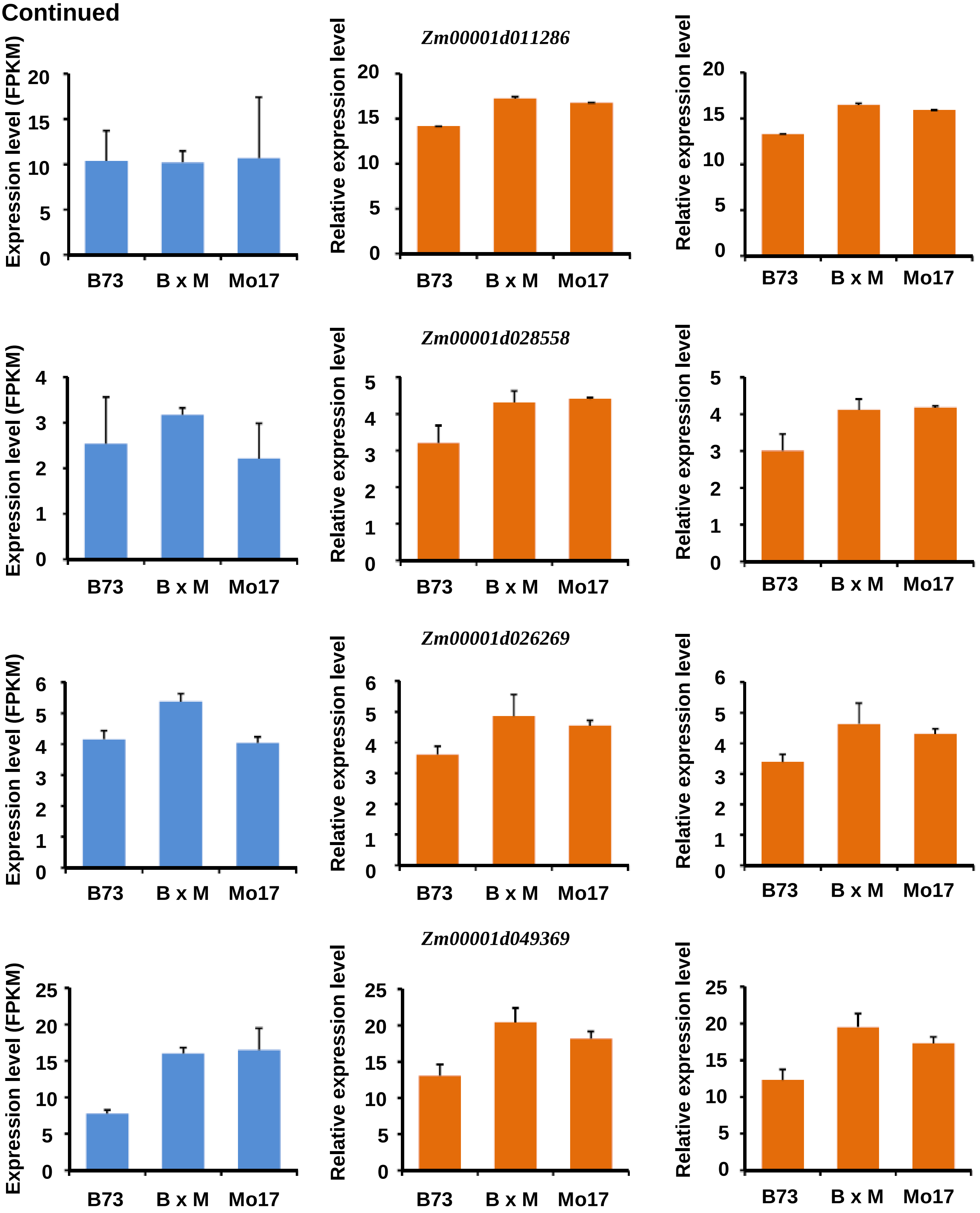

Figure S2. RT-qPCR validation of eight genes at the mRNA level in F_1 hybrid B73×Mo17 and its two parental lines B73 and Mo17. The mRNA levels were determined by RNA-seq (blue) or RT-qPCR (red), respectively. The relative mRNA level of each gene was compared to the internal control genes *Zm00001d034600* (middle) and *Zm00001d042939* (right). Data are represented as means + s.d of two independent biological replicates.

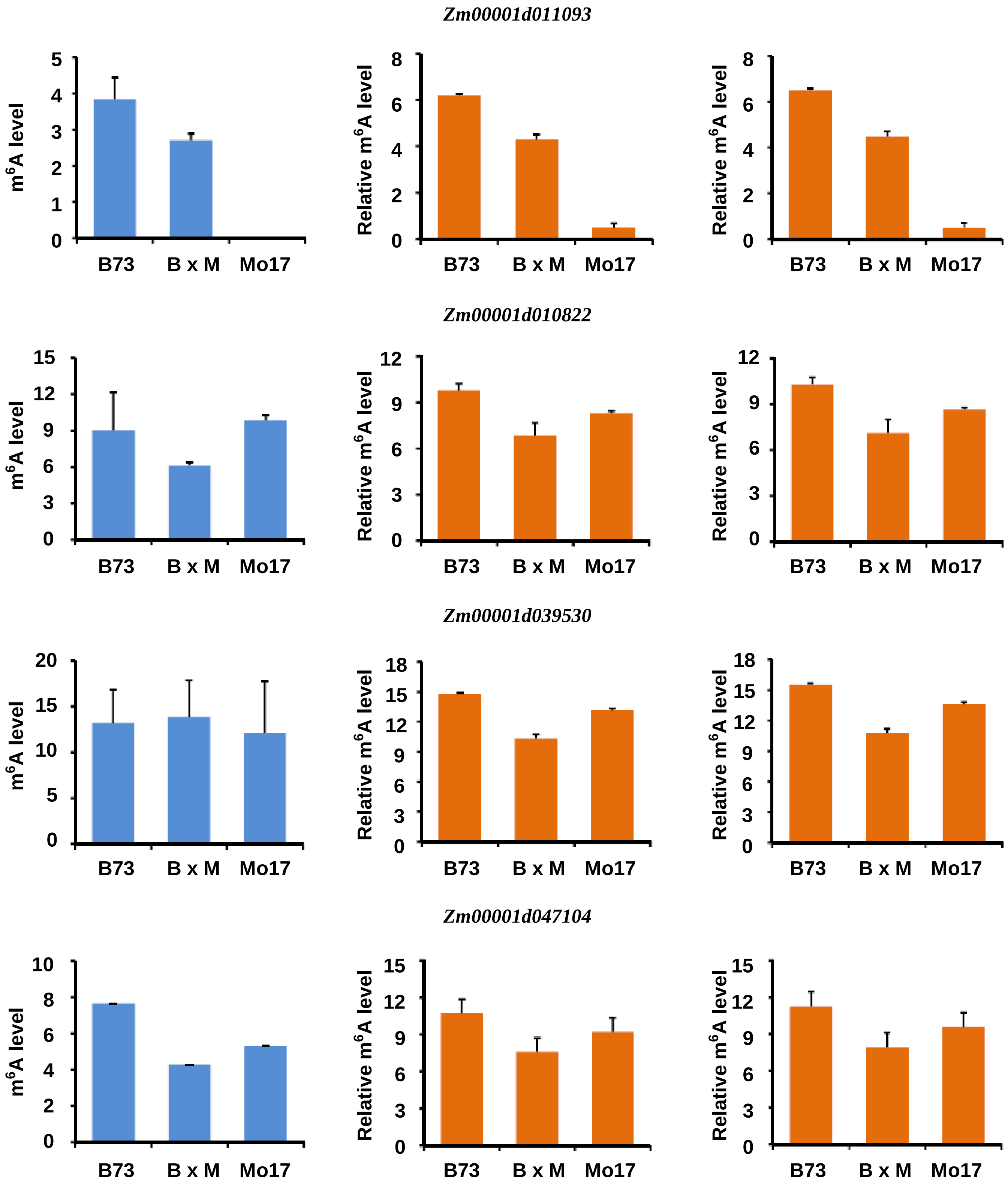

Zm00001d011093
m6A level
Relative m6A level
Relative m6A level
B73
B x M
Mo17
Zm00001d010822
m6A level
Relative m6A level
Relative m6A level
B73
B x M
Mo17
Zm00001d039530
m6A level
Relative m6A level
Relative m6A level
B73
B x M
Mo17
Zm00001d047104
m6A level
Relative m6A level
Relative m6A level
B73
B x M
Mo17

Continued

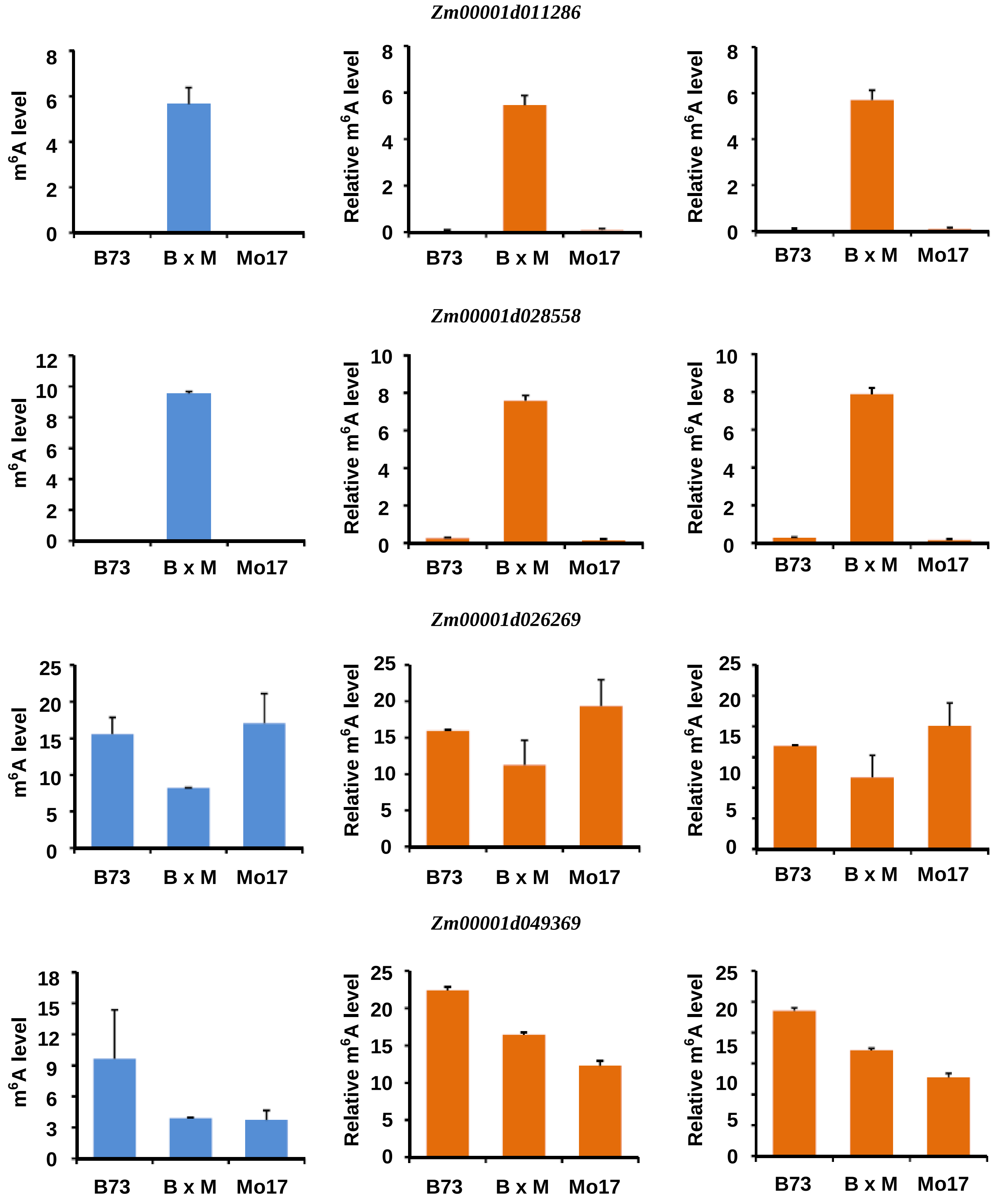

Figure S3. RT-qPCR validation of eight genes at the m^{6}A level in F$_1$ hybrid B73×Mo17 and its two parental lines B73 and Mo17. The m^{6}A levels determined by m^{6}A-seq (blue) or RT-qPCR (red), respectively The relative m^{6}A level of each gene was compared to the internal control genes *Zm00001d034600* (middle) and *Zm00001d042939* (right). Data are represented as means + s.d of two independent biological replicates.

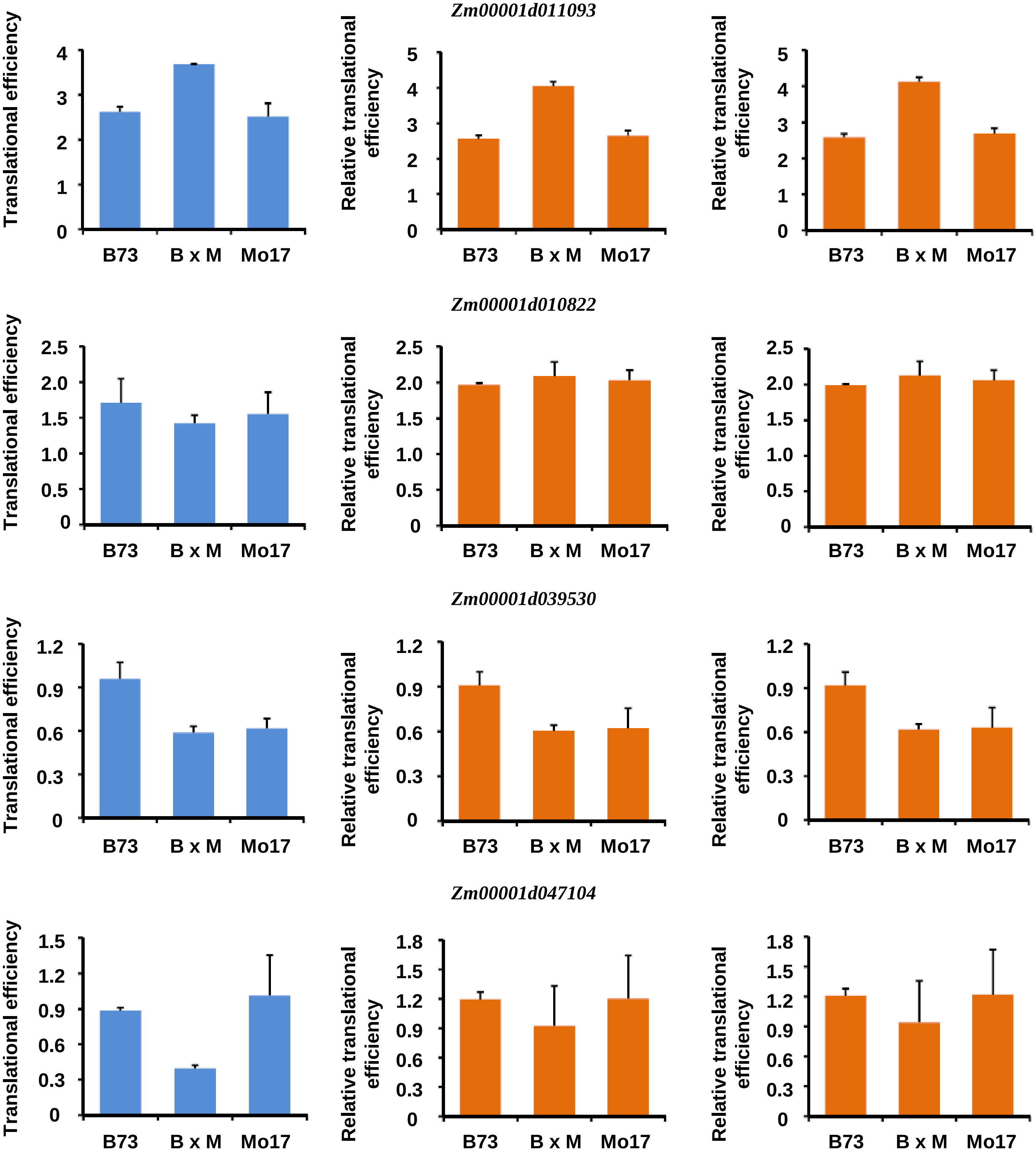
Zm00001d011093
Zm00001d010822
Zm00001d039530
Zm00001d047104
Translational efficiency
Relative translational efficiency
B73
B x M
Mo17

Continued

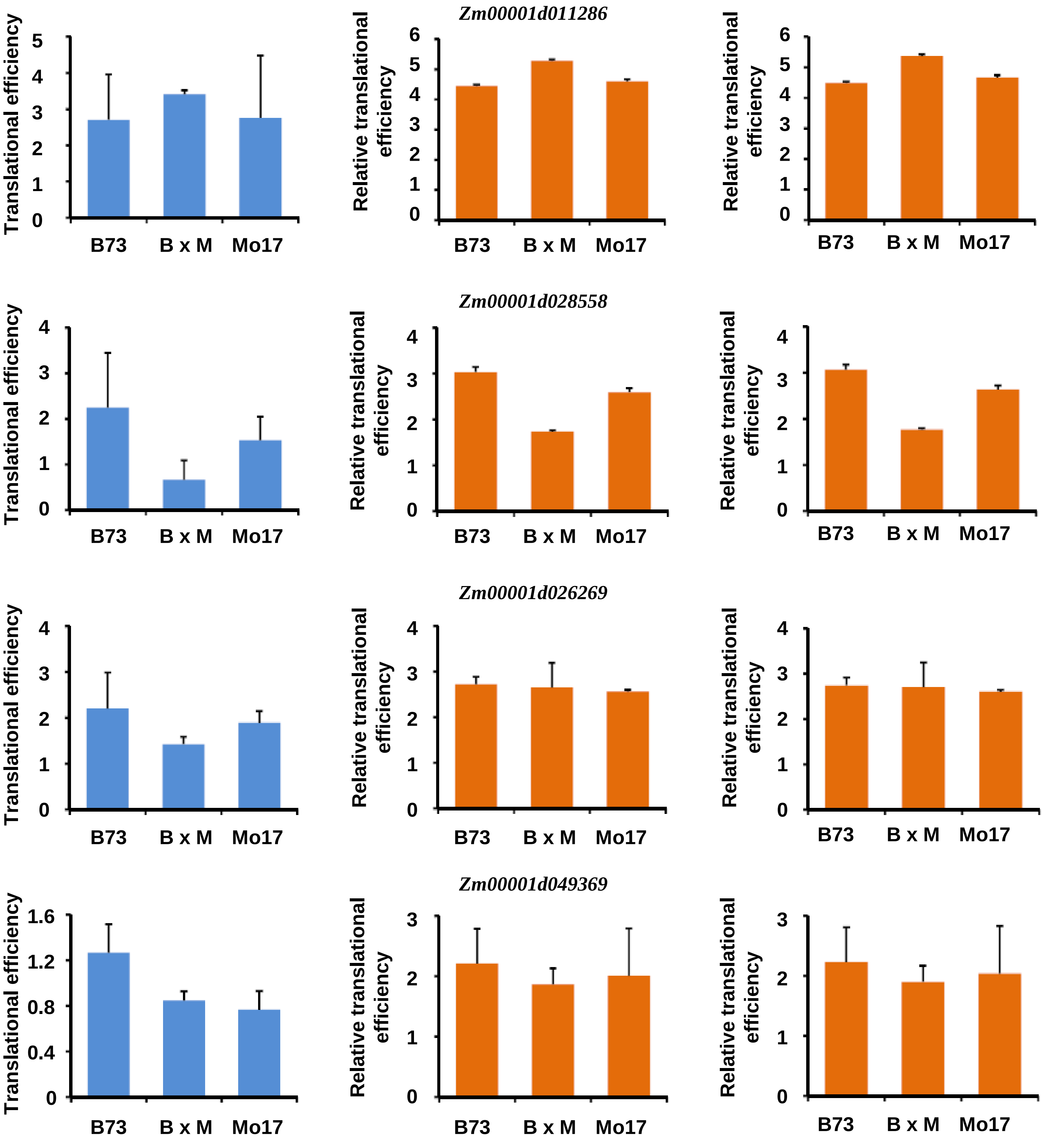

Figure S4. RT-qPCR validation of selected eight genes at the level of translational efficiency in F_1 hybrid B73×Mo17 and its two parental lines B73 and Mo17. The translational efficiency was determined by polysome profiling sequencing (blue) or RT-qPCR (red), respectively. The relative translational efficiency of each gene was compared to the internal control genes *Zm00001d034600* (middle) and *Zm00001d042939* (right). Data are represented as means + s.d of two independent biological replicates.

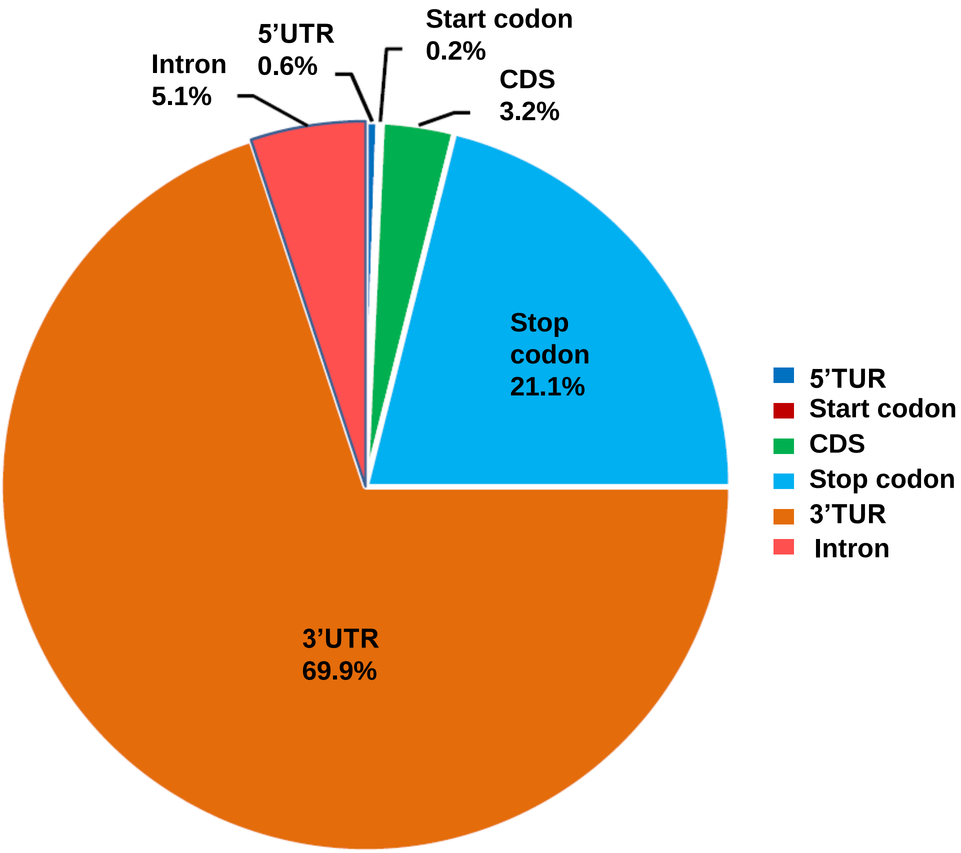

Figure S5. Pie-chart depicting the percentage of m^6A peaks within six transcript segments in hybrid.

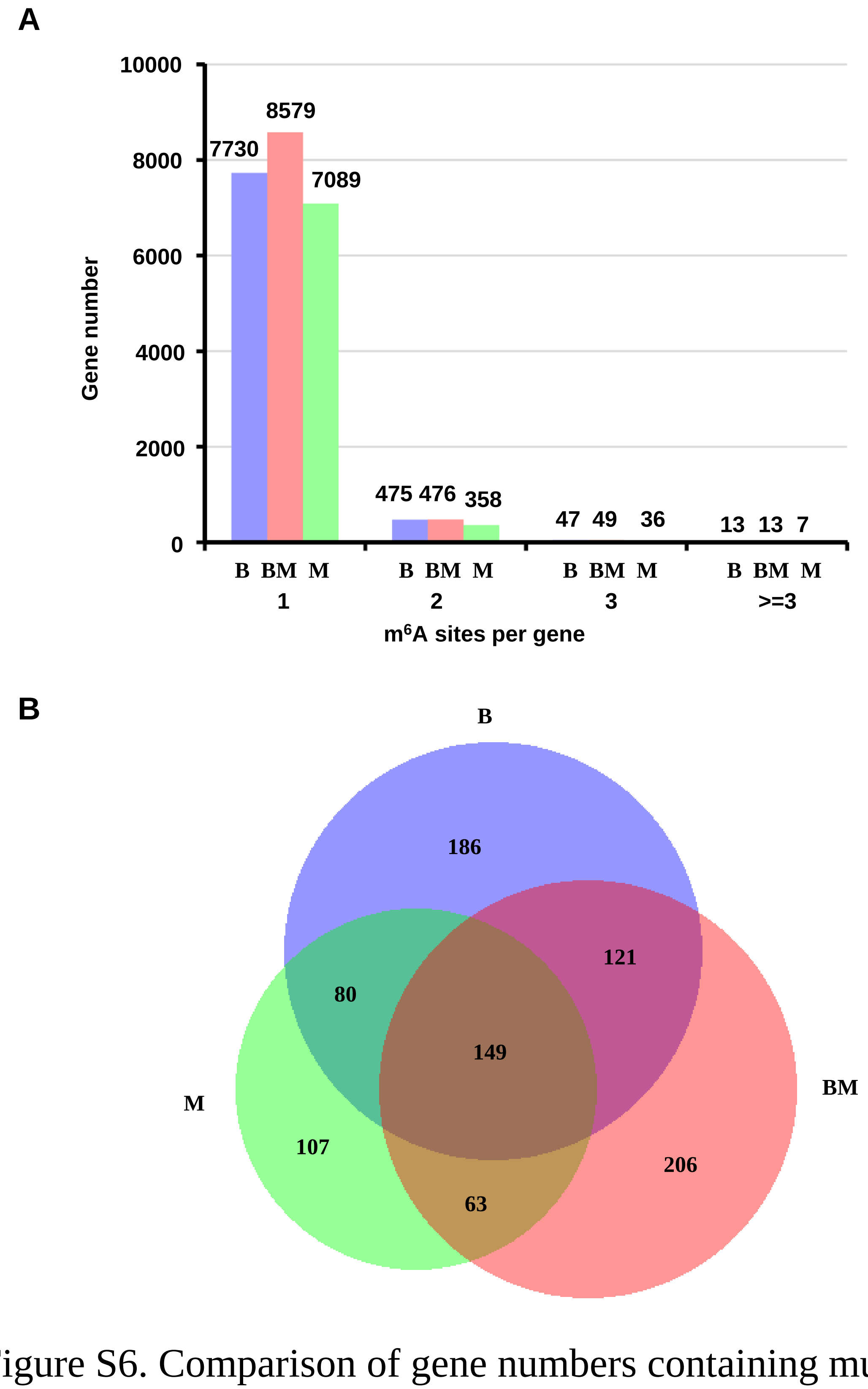

Figure S6. Comparison of gene numbers containing multiple m^6A peaks between hybrid and parents.

(A) No. of genes containing different number of m^6A peak between hybrid and parents.

(B) Venn diagram showing the overlap of genes containing multiple m^6A peak per gene between hybrid and parents. B, B73; M, Mo17; BM, B73 x Mo17.
